# Contribution of common chronic conditions to midlife physical function decline: The Study of Women’s Health Across the Nation

**DOI:** 10.1186/s40695-020-00053-0

**Published:** 2020-07-28

**Authors:** Brittney S. Lange-Maia, Kelly Karavolos, Elizabeth F. Avery, Elsa S. Strotmeyer, Carrie A. Karvonen-Gutierrez, Bradley M. Appelhans, Imke Janssen, Sheila A. Dugan, Howard M. Kravitz

**Affiliations:** 1 Department of Preventive Medicine, Rush University Medical Center, Chicago, IL USA; 2 Center for Community Health Equity, Rush University Medical Center, Chicago, IL USA; 3 Department of Epidemiology, Graduate School of Public Health, University of Pittsburgh, Pittsburgh, PA USA; 4 Department of Epidemiology, School of Public Health, University of Michigan, Ann Arbor, MI USA; 5 Department of Physical Medicine & Rehabilitation, Rush University Medical Center, Chicago, IL USA; 6 Department of Psychiatry and Behavioral Sciences, Rush University Medical Center, Chicago, IL USA

**Keywords:** Chronic conditions, Disability, Aging, Physical function, Incident rate ratio

## Abstract

**Background:**

Chronic conditions are associated with worse physical function and commonly develop during midlife. We tested whether the presence of 8 chronic conditions, or the development of these conditions, is associated with declines in physical function among midlife women as they transition into early late life.

**Methods:**

Participants (*N* = 2283) were from the Study of Women’s Health Across the Nation. Physical function was assessed at 8 visits starting at the study’s fourth clinic visit in 2000/2001 through follow-up visit 15 (2015/2017) using the Short Form-36 Physical Function subscale. Chronic conditions included diabetes, hypertension, osteoarthritis, osteoporosis, stroke, heart disease, cancer, and depressive symptoms. Repeated-measures Poisson regression modeled associations between 1) prevalent chronic conditions at analytic baseline (visit 4) and longitudinal physical function, and 2) change in physical function associated with developing a new condition. Models were adjusted with the total number of other chronic conditions at visit 4.

**Results:**

In separate fully-adjusted longitudinal models, prevalent heart disease and osteoporosis were associated with 18% (IRR = 0.815, 95% confidence interval [CI]: 0.755–0.876) and 12% (IRR = 0.876, 95% CI: 0.825–0.927) worse initial physical function, respectively. Prevalent osteoarthritis was associated with approximately 6% (IRR = 0.936, 95% CI: 0.913–0.958) worse initial physical function, and a slight additional worsening over time (IRR = 0.995, 95% CI: 0.994–0.996). A 12% (IRR = 0.878, 95% CI: 0.813–0.950) decrease in physical function concurrent with stroke development was evident, as was accelerated decline in physical function concurrent with heart disease development (IRR = 0.991, 95% CI: 0.988–0.995).

**Conclusions:**

Initial prevalent conditions related to the musculoskeletal system were associated with worse initial physical function, with some evidence of accelerated decline in physical function with osteoarthritis. Stroke and heart disease are less common than osteoarthritis in this age group, but the severe effects of these conditions on physical function shows the need for a greater focus on cardiovascular health during midlife. Women who develop chronic conditions during midlife may be at particular risk for poor physical function as they age, warranting disability prevention efforts focused on this population.

## Background

Though much is known about chronic conditions and physical function in late life, the relationship of chronic condition development in midlife with subsequent physical function change and disability is less clear. In late life chronic health conditions and comorbidities are strongly related to worse physical function and greatly contribute to disability [1–3]. Among older adults who develop one new condition, the odds of disability onset are twice as high within one year, and up to 13 times higher among those who develop three or more conditions compared to older adults who do not develop chronic conditions [4]. Chronic conditions do not influence physical function uniformly, as previous studies have identified musculoskeletal diseases, stroke, clinically-meaningful depressive symptoms, and cardiovascular disease having some of the greatest contributions to disability among community-dwelling older adults [2, 5, 6].

Midlife is a critical period in the life course as it is when chronic conditions tend to increase and declines in physical function become apparent; and for women, is accompanied by hormonal changes due to the menopausal transition. Much of the work regarding chronic conditions and physical function in midlife has focused on prevalent chronic conditions [7–9]. However, less is known regarding how physical function changes in conjunction with the development of new chronic conditions among populations transitioning from midlife to late life. Midlife is also a critical period from a prevention standpoint as midlife health and behaviors are strongly related to physical function into late life [10, 11].

Previously, we demonstrated that the development of one or multiple new chronic conditions among midlife women was associated with accelerated decline in self-reported physical function among midlife women [12]. However, the degree to which specific existing or new chronic conditions drove that physical function decline in women is the focus of this current study. We hypothesized that different chronic conditions contribute to physical function decline differentially in midlife. In particular, we hypothesized that conditions with multiple/intense symptoms (i.e. heart disease, stroke, cancer, diabetes), or that may have been diagnosed due to reporting difficulties in physical function (i.e. osteoarthritis), would be associated with greater declines in physical function associated with their development whereas other conditions (depressive symptoms, osteoporosis, hypertension) would be associated with gradual declines over time.

## Methods

### Participants

Participants were from the fourth annual follow-up visit (2000/01) of the Study of Women’s Health Across the Nation (SWAN), a longitudinal, multi-ethnic cohort study of health during the menopausal transition. Descriptions of the study design and recruitment have been previously published [13]. In 1996/97, SWAN recruited 3302 women, age 42–52 years, from seven U.S. clinical sites (Boston, MA, Chicago, IL, southeastern Michigan, Los Angeles, CA, Newark NJ, Oakland, CA, and Pittsburgh, PA). Women were eligible for the study if they had an intact uterus and at least one ovary, were premenopausal or early peri-menopausal (i.e., had at least one menstrual period in the past 3 months), had not used reproductive hormones in the previous 3 months, and were not pregnant, lactating or breastfeeding. The fourth follow-up visit (occurring in 2000/2001) served as the analytic baseline visit for this study. Consistent with previous work examining the effect of prevalent and new chronic conditions on physical function trajectories [12], women were included in this analysis if they had complete data on physical function at the fourth follow-up visit in 2000/01 and at least two additional physical function assessments through visit 15 in 2016/2017. Follow-up visits were conducted annually or biennially, with a mean follow-up of 14.0 ± 2.6 years (min-max: 3.4–16.8 years). In total, 59.8% of participants had data on all 8 of the included study visits, and 95.2% of included participants had data on at least half of the visits (4 visits or more).

### Self-reported physical function

Physical function was assessed via self-report using the 10-item physical function subscale of the Medical Outcomes Study Short Form 36 (SF-36) [14]. This scale assesses the level of limitation (“limited a lot”, “limited a little”, or “not limited at all”) in ten common activities and was included at annual follow-up visits 4, 6, 8, 10, 11, 12, 13, and 15. Scores were transformed to range from 0 to 100; higher scores indicate better physical function.

### Chronic conditions

Chronic conditions were selected based upon prior literature indicating their relationships with physical function in mid- or late life women [7, 15–18], including work from the SWAN cohort, as previously described [12]. All chronic conditions were assessed via self-report of a healthcare provider diagnosis (with the exception of clinically-meaningful depressive symptoms) and included diabetes, hyptertension, osteoarthritis, osteoporosis, stroke, heart disease (myocardial infarction or angina), and cancer (excluding skin). Depression was not assessed via self-reported healthcare diagnosis; instead, we used the Center for Epidemiologic Studies Depression (CES-D) scale (20 items, scores can range from 0 to 60), with clinically-meaningful depression symptoms defined as a score of ≥16 [19]. Chronic conditions were collected at all follow-up visits 4–15, inclusive. Because physical function was assessed at fewer visits, chronic conditions identified at visits at which physical function was not assessed were carried forward to the next concurrent visit. These conditions are indicative of dysfunction in a variety of bodily systems (including musculoskeletal and cardiovascular), and can range in severity and intensity of their development.

### Covariates

All covariates were from the fourth clinic visit (analytic baseline) unless specified otherwise. Sociodemographic characteristics included age, race/ethnicity (non-Hispanic white, African American, Chinese, Hispanic, Japanese), financial strain (difficulty paying for basics), marital status, and educational attainment (all collected at SWAN baseline aside from marital status). Self-rated health (“excellent,” “very good,” “good,” “fair,” and “poor”), physical activity (using the Kaiser Physical Activity Survey, KPAS, collected at Visit 3, or Visit 5 if missing from Visit 3) [20] smoking status, hormone use, bodily pain (using the SF-36 Bodily Pain Subscale), and fractures were assessed via self-report. Menopausal status was determined using bleeding criteria and categorized as premenopausal, early peri-menopausal, late peri-menopausal, and postmenopausal. Women reporting a hysterectomy with or without bilateral oophorectomy were classified as “surgically menopausal,” and pre- and perimenopausal women whose status could not be determined due to hormone therapy, were classified as unknown. Menopausal status and hormone use were included as time-varying covariates. Body mass index was calculated using measured height and weight.

### Statistical methods

We compared initial participant characteristics between women who 1) had no chronic conditions at follow-up visit 4 and throughout the study follow up, 2) developed a condition after follow-up visit 4 (no initial prevalent conditions), and 3) had an initial prevalent condition at follow-up visit 4. Continuous variables were compared using ANOVA (normally-distributed variables) or Kruskal-Wallis (non-normally distributed variables) tests as appropriate and chi-squared tests for categorical measures.

The outcome, self-reported physical function, followed a Poisson distribution. Thus, we used repeated measures Poisson regression models to assess the associations between chronic conditions (both prevalent and new (incident) chronic conditions) and longitudinal physical function. Beta estimates from these models were exponentiated to yield incident rate ratios (IRRs). These IRRs are interpreted as the score someone with a one-unit increase in any of the model parameters (for example, having a specific condition) would have in comparison to someone without that unit increase (for example, not having that condition). IRRs can also be subtracted from 1 and multiplied by 100 to obtain a percent difference rather than a ratio. We retained three decimal places for the IRRs in order to better interpret the percent differences, particularly as our previous research has indicated that these effects may be small on an annual basis [12].

To determine the association between a prevalent chronic condition and longitudinal change in physical function, the models included a main effect for the baseline condition (to detect an initial difference in physical function at analytic baseline) and interaction with time (to detect a difference in physical function change over time). Next, to determine whether physical function changed differently after the development of a new chronic condition, we included an inflection point at the time that a new chronic condition was identified (to detect whether physical function changed concurrently with the new condition), and interaction between the new condition and time (to detect a difference in physical function change over time). Only participants without that condition at follow-up visit 4 were included in these models. For example when examining diabetes, the sample included women who did not have diabetes at the analytic baseline. The inflection point was based on the visit at which women first noted to have diabetes.

Each condition was modeled separately, and we adjusted for the total number of other conditions from follow-up visit 4. All models were adjusted for covariates from the fourth follow-up visit, including age, race/ethnicity, financial strain, marital status, education, hormone use, smoking, body mass index, health status, bodily pain, fracture history, and physical activity.

## Results

Table 1 shows that the vast majority (*N* = 1604, 70.3%) of the 2283 women included in the analytic sample had at least one chronic condition at follow-up visit 4. Among the women with no conditions at follow-up visit 4 (*N* = 679), most (*N* = 513) developed a new condition during follow up, leaving only 7.3% (*N* = 166) of the original sample free of chronic conditions during their follow-up within the study. Women who had chronic conditions at follow-up visit 4 tended to be older, were more often African American or Hispanic, had lower levels of education, were more likely to be separated, widowed, or divorced, and were more likely to be at later stages of the menopausal transition compared to women who did not have chronic conditions at follow-up visit 4 (*p* < 0.001 for each participant characteristic; Table 1). Women with chronic conditions at follow-up visit 4 also reported worse overall health, had higher body mass index, were more likely to smoke, reported more bodily pain and were less physically active. Many of these characteristics followed a gradient pattern such that women who remained condition-free had better health compared to women who developed conditions during follow-up, with women with conditions at follow-up visit 4 having the worst health indicators. There was no difference in fracture history between groups. Physical function was highest among women who had no conditions through follow-up (median initial score 100, interquartile range 95–100), and women with follow-up visit 4 conditions had the worst (median initial score 90, interquartile range 70–100).

**Table 1:** Participant characteristics at analytic baseline (follow-up visit 4) by chronic condition status

Participant Characteristics	All Participants N = 2283	No Chronic Conditions through Follow-Up *N* = 166	Developed Condition During Follow-Up *N* = 513	Has Prevalent Chronic Condition^a^ *N* = 1604	*P*-Value
Age, years, mean ± SD^b^	50.0 ± 2.7	49.5 ± 2.6	49.8 ± 2.7	50.1 ± 2.7	0.003
Race/Ethnicity, n (%)					<.0001
Non-Hispanic White	1122 (49.2)	91 (54.8)	273 (53.2)	758 (47.3)	
African American	593 (26.0)	24 (14.5)	92 (17.9)	477 (29.7)	
Hispanic	117 (5.1)	5 (3.0)	13 (2.5)	99 (6.2)	
Chinese	209 (9.2)	26 (15.7)	62 (12.1)	121 (7.5)	
Japanese	242 (10.6)	20 (12.1)	73 (14.2)	149 (9.3)	
Education, n (%)					<.0001
High school or less	470 (20.7)	26 (15.8)	85 (16.7)	359 (22.6)	
Some college	736 (32.5)	43 (26.1)	149 (29.3)	544 (34.2)	
College degree	491 (21.7)	44 (26.7)	119 (23.4)	328 (20.6)	
Post college	569 (25.1)	52 (31.5)	156 (30.7)	361 (22.7)	
Financial strain, any, n (%)	785 (35.1)	32 (19.4)	124 (24.8)	629 (40.1)	<.0001
Marital status, n (%)					<.0001
Single/never married	307 (13.5)	23 (13.9)	54 (10.6)	230 (14.4)	
Married/living as married	1480 (65.1)	128 (77.1)	370 (72.6)	982 (61.5)	
Separated/Widowed/Divorced	486 (21.4)	15 (9.0)	86 (16.9)	385 (24.1)	
Menopausal Status, n (%)					0.0002
Premenopausal	162 (7.1)	19 (11.5)	45 (8.8)	98 (6.1)	
Early peri-menopausal	1017 (44.7)	82 (49.4)	258 (50.6)	677 (42.4)	
Late peri-menopausal	231 (10.2)	14 (8.4)	42 (8.2)	175 (11.0)	
Postmenopausal	465 (20.5)	34 (20.5)	78 (15.3)	353 (22.1)	
Surgical menopause	62 (2.7)	1 (0.6)	13 (2.6)	48 (3.0)	
Unknown status	336 (14.8)	16 (9.6)	74 (14.5)	246 (15.4)	
Self-rated health status, n (%)					<.0001
Excellent	367 (16.3)	65 (39.2)	142 (28.1)	160 (10.1)	
Very Good	858 (38.0)	72 (43.4)	231 (45.7)	555 (35.0)	
Good	724 (32.1)	26 (15.7)	113 (22.3)	585 (36.9)	
Fair	270 (12.0)	3 (1.8)	20 (4.0)	247 (15.6)	
Poor	39 (1.7)	0 (0.0)	0 (0.0)	39 (2.5)	
BMI, kg/m^2^, mean ± SD	28.8 ± 7.4	24.9 ± 4.2	26.2 ± 5.8	30.0 ± 7.7	<.0001
Current smoker, n (%)	306 (13.4)	9 (5.4)	57 (11.2)	240 (15.0)	0.0006
Bodily pain^c^, median (q1, q3)	72 (51, 84)	84 (74, 100)	84 (62, 84)	62 (51, 84)	<.0001
Fracture history, n (%)	39 (1.7)	2 (1.2)	7 (1.4)	30 (1.9)	0.651
KPAS^d^ score, mean ± SD	7.7 ± 1.8	8.2 ± 1.7	8.0 ± 1.7	7.5 ± 1.8	<.0001
Physical function^e^, median (q1, q3)	90.0 (75.0, 100.0)	100 (95, 100)	95 (90,100)	90 (70, 100)	<.0001

Note: ^a^Of the women with baseline chronic conditions, 1087 also developed another during follow-up. ^b^Standard deviation; ^c^ SF-36 Bodily Pain subscale, scores range from 0 to 100, ^d^Kaiser Physical Activity Survey; ^e^SF-36 Physical Function subscale, scores range from 0 to 100

At follow-up visit 4, the most common conditions were clinically-meaningful depressive symptoms (44.6%), osteoarthritis (30.5%), and hypertension (30.4%), while diabetes (7.1%), osteoporosis (4.2%), cancer (3.7%), heart disease (3.0%), and stroke (1.3%) were far less common (Table 2). Clinically-meaningful depressive symptoms, hypertension, osteoarthritis, and osteoporosis had the highest prevalence throughout follow-up. Overall, clinically-meaningful depressive symptoms had the shortest median time until development (4.8 years) whereas cancer had the longest (9.7 years). The other conditions developed after a median of 6.5 to 7.8 years of follow-up.

**Table 2:** Individual Chronic Conditions, initial and follow-up status

Chronic Condition	Women with prevalent condition at analytic baseline (follow-up visit 4) N (%)	Women who developed condition during follow-up N (%)	Time Before Condition Developed Median Years (Interquartile Range)
Depressive Symptoms^a^	958 (44.6)	316 (13.8)	4.8 (2.1–7.6)
Osteoarthritis	697 (30.5)	714 (31.3)	6.5 (4.0–11.0)
Hypertension	693 (30.4)	580 (25.4)	6.9 (4.0–10.3)
Diabetes	163 (7.1)	307 (13.5)	7.5 (4.0–10.4)
Osteoporosis	96 (4.2)	471 (20.6)	7.8 (5.6–11.3)
Cancer	84 (3.7)	212 (9.3)	9.7 (6.0–14.6)
Heart Disease	68 (3.0)	107 (4.7)	6.6 (4.0–9.9)
Stroke	30 (1.3)	60 (2.6)	7.8 (5.1–11.4)

^a^Clinically relevant depressive symptoms, defined as Center for Epidemiologic Studies Depression Scale score of ≥16 points

In separate, fully adjusted longitudinal models, examining the association between initial prevalent chronic conditions and physical function, heart disease and osteoporosis were associated with about 18% (IRR = 0.815, 95% confidence interval [CI]: 0.754–0.876) and 12% (IRR = 0.876, 95% CI: 0.825–0.927) worse initial physical function, respectively (Table 3). Prevalent osteoarthritis was associated with approximately 6% (IRR = 0. 936, 95% CI: 0.913–0.958) worse initial physical function, and about an additional 0.5% (IRR = 0.995, 95% CI: 0.994–0.996) worsening every year compared to those without osteoarthritis, though this additional slowing was only borderline significant. We also saw faster decline in physical function over time associated with clinically-meaningful depressive symptoms, hypertension, diabetes, and stroke, though these effects were small (ranging from − 0.8% to − 0.4% per year) and only borderline statistically significant. We observed no difference in either initial physical function or change in physical function with midlife cancer.

**Table 3:** Effect of specific prevalent chronic conditions on longitudinal physical function

Model Parameters	Clinically-Meaningful Depressive Symptoms^a^		Osteoarthritis		Hypertension		Diabetes	
	IRR^**b**^	95% CI	IRR	95% CI	IRR	95% CI	IRR	95% CI
Number of Other Conditions	0.933	0.922, 0.944	0.954	0.943, 0.966	0.934	0.923, 0.945	0.941	0.931, 0.951
Time in Years	0.996	0.996, 0.997	0.996	0.995, 0.996	0.996	0.995, 0.996	0.995	0.994, 0.995
Condition of Interest	1.000	0.979, 1.021	0.936	0.913, 0.958	1.006	0.984, 1.029	0.990	0.951, 1.029
Condition of Interest x Time	0.996	0.995, 0.966	0.995	0.994, 0.996	0.995	0.994, 0.996	0.994	0.992, 0.996
Model Parameters	**Osteoporosis**		**Cancer**		**Heart Disease**		**Stroke**	
	**IRR**	**95% CI**	**IRR**	**95% CI**	**IRR**	**95% CI**	**IRR**	**95% CI**
Number of other conditions	0.947	0.937, 0.957	0.941	0.931, 0.950	0.951	0.942, 0.961	0.945	0.935, 0.954
Time in Years	0.994	0.994, 0.995	0.995	0.994, 0.995	0.995	0.994, 0.995	0.995	0.994, 0.995
Condition of Interest	0.876	0.825, 0.927	0.985	0.932, 1.037	0.815	0.754, 0.876	0.911	0.817, 1.006
Condition of Interest x Time	1.001	0.999, 1.004	1.001	0.999, 1.003	0.998	0.995, 1.001	0.992	0.987, 0.997

Note: Models were adjusted for covariates, including age, race/ethnicity, financial strain, marital status, education, menopausal status, hormone use, smoking, body mass index, health status, bodily pain, fracture history, and physical activity. ^a^Clinically relevant depressive symptoms, defined as Center for Epidemiologic Studies Depression Scale score of ≥16 points. ^b^IRR: Incident rate ratio. To obtain percent differences in physical function associated with each model parameter, subtract the IRR from 1 and multiply by 100

**Fig. 1:**
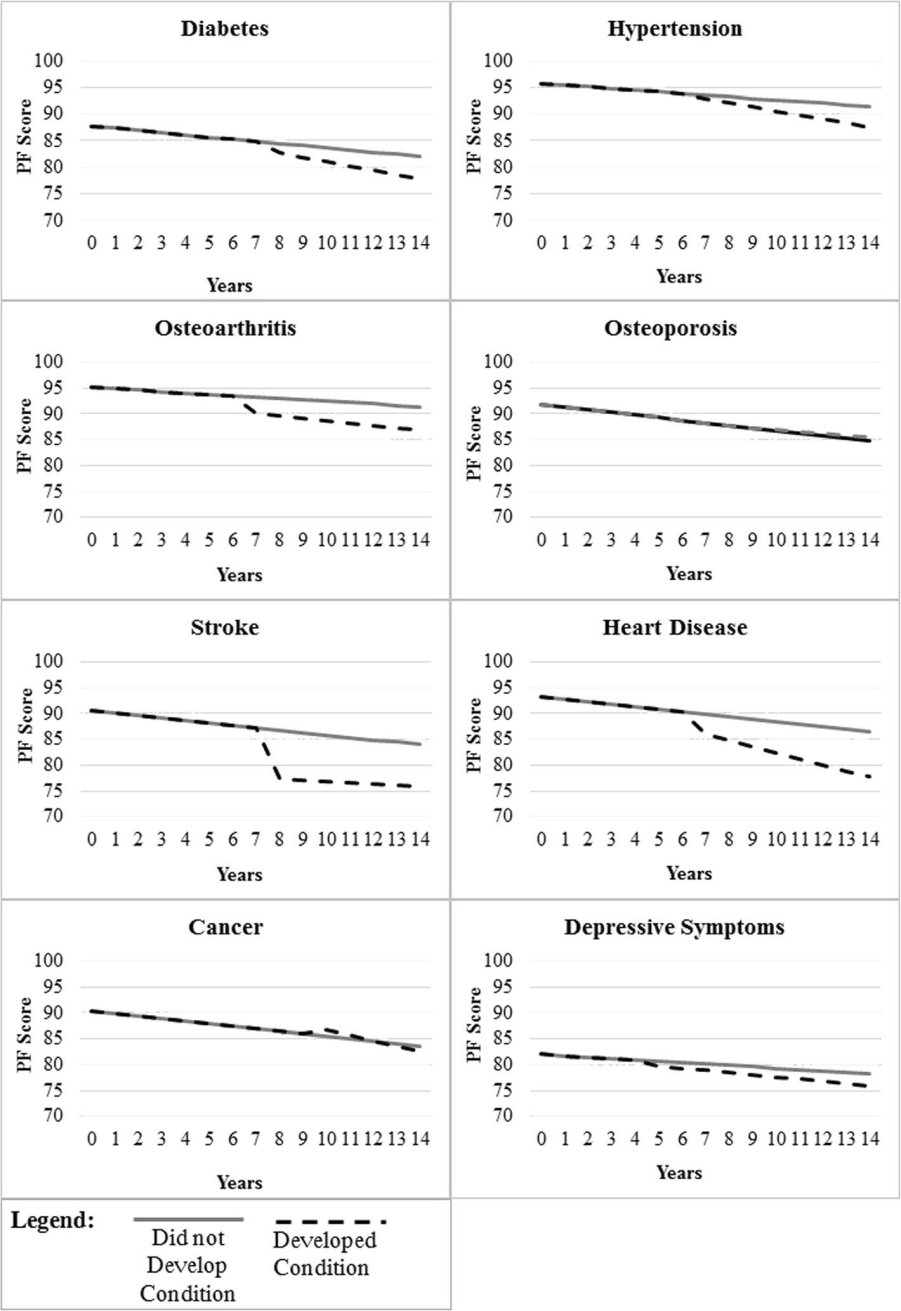
Change in physical function with the development of a new condition. Note: Solid grey lines represent women who did not develop the condition of interest, while the dashed black lines represent women who did. Sample sizes vary between models because only women without the condition at baseline were included. The change-point for each model was set at the median time-point when the condition was developed. Depressive symptoms indicate clinically relevant depressive symptoms, defined as Center for Epidemiologic Studies Depression Scale score of ≥16 points. Changes in physical function in conjunction with chronic condition development were statistically significant (drop in physical function and/or change in slope) for all conditions except for depressive symptoms and osteoporosis (see Table 4)

In separate, fully-adjusted longitudinal models examining the association between each new chronic condition and change in physical function, women who reported having a stroke had about a 12% (IRR = 0.881, 95% CI: 0.813–0.950) drop in physical function at the visit where it was first reported (Table 4). We observed accelerated decline in physical function associated with the development of diabetes (IRR = 0.995, 95% CI: 0.992–0.997) and heart disease (IRR = 0.991, 95% CI: 0.988–0.995), though these effects were only borderline significant. For osteoarthritis, relationships showing an accelerated decline in physical function (IRR = 0.997, 95% CI: 0.996–0.999) after its development reached borderline statistical significance. For hypertension and cancer, there was an apparent small increase in physical function associated with the development of these conditions. However, when considering both the main effect and interaction with time; this slight increase was no longer noticeable, as shown in Fig. 1. Similarly, though the term for the main effect of diabetes did not indicate a decrease in physical function concurrent with the development of diabetes (the confidence interval included 1.0), a statistically significant decrement over time is evident due to the interaction term as the slope change associated with diabetes development starts at that visit, rather than only changing after. We saw no effect on physical function associated with the development of osteoporosis.

**Table 4:** Change in Self-Reported Physical Function Concurrent with the Development of New Chronic Conditions

Model Parameters	Clinically-Meaningful Depressive Symptoms^a^		Osteoarthritis		Hypertension		Diabetes	
	IRR^**b**^	95% CI	IRR	95% CI	IRR	95% CI	IRR	95% CI
Number of Baseline Conditions	0.936	0.923, 0.949	0.974	0.963, 0.985	0.950	0.939, 0.961	0.946	0.936, 0.956
Time in Years	0.996	0.995, 0.997	0.998	0.997, 0.998	0.997	0.996, 0.997	0.996	0.995, 0.996
New Condition	0.995	0.975, 1.015	0.986	0.970, 1.002	1.033	1.015, 1.050	1.022	0.996, 1.048
New Condition x Time	0.998	0.996, 1.000	0.998	0.997, 0.999	0.995	0.993, 0.996	0.995	0.992, 0.997
Model Parameters	**Osteoporosis**		**Cancer**		**Heart Disease**		**Stroke**	
	**IRR**	**95% CI**	**IRR**	**95% CI**	**IRR**	**95% CI**	**IRR**	**95% CI**
Number of Baseline Conditions	0.950	0.940, 0.959	0.942	0.932, 0.951	0.955	0.945, 0.964	0.946	0.936, 0.955
Time in Years	0.994	0.994, 0.995	0.995	0.994, 0.995	0.995	0.994, 0.995	0.995	0.994, 0.995
New Condition	0.990	0.969, 1.012	1.090	1.053, 1.126	1.014	0.971, 1.056	0.881	0.813,0.950
New Condition x Time	1.001	0.999, 1.003	0.993	0.990, 0.996	0.991	0.984, 0.995	1.002	0.996, 1.008

Note: Sample sizes vary between models because only women without the condition at analytic baseline (follow-up visit 4) were included. Models were adjusted for covariates, including age, race/ethnicity, financial strain, marital status, education, menopausal status, hormone use, smoking, body mass index, health status, bodily pain, fracture history, and physical activity. ^a^Clinically relevant depressive symptoms, defined as Center for Epidemiologic Studies Depression Scale score of ≥16 points. ^b^IRR: Incident rate ratio. To obtain percent differences in physical function associated with each model parameter, subtract the IRR from 1 and multiply by 100

## Discussion

In this study of midlife women, existing and new chronic conditions were common, and these prevalent conditions, as well as the development of new conditions were associated with decreases in physical function over time. This study included a variety of types of chronic conditions, both in terms of the bodily systems they impact and in terms of the intensity of their symptoms and treatment. Very few women (7.3%) remained free of chronic conditions during the duration of this study as they transitioned from midlife to early late life, highlighting the importance of midlife regarding primary and secondary chronic disease prevention during this timeframe. Further, we determined the degree to which specific conditions contribute to this decline and in midlife, with prevalent osteoarthritis, osteoporosis, and heart disease each being associated with worse initial physical function, and newly developed stroke being associated with a drop in physical function. Several initial prevalent conditions were associated with accelerated decline in physical function, including clinically-meaningful depressive symptoms, osteoarthritis, hypertension, diabetes, and stroke, however, these effects were small overall. Newly developed osteoarthritis, hypertension, diabetes, cancer, and heart disease were also associated with accelerated declines in physical function, but again, these effects were small.

Osteoarthritis was one of the most prevalent conditions at follow-up visit 4 (approximately 31%) and was one of the most commonly developed conditions thereafter, with 31% of the women initially free of osteoarthritis ultimately developing it. Osteoarthritis was the only condition that was related to worse initial physical function and accelerated decline after development. Previous work in SWAN has shown that midlife women with osteoarthritis have approximately 2.8 times higher odds of mobility disability (moderate, severe, or extreme problems with mobility), even when accounting for obesity [17]. Disability in relation to osteoarthritis tends to follow a slow progression pattern rather than fast onset catastrophic disability, emphasizing the need for early interventions in order to slow its progression once developed in order to delay disability [21].

Stroke and heart disease were the two least common conditions among our sample of midlife women, however, the strong effects of these conditions on physical function shows the need for a greater focus on cardiovascular health during the midlife period regarding physical function. Hypertension, another important aspect of cardiovascular health was much more common compared to overt cardiovascular disease and stroke but was still associated with accelerated decline in physical function. These accelerations in physical function decline were evident in both prevalent and incident hypertension and were somewhat surprising given that hypertension generally has few symptoms that would be thought of as influencing self-reported physical function at these younger ages. Previous studies have shown faster declines in gait speed, activities of daily living, and other aspects of physical function among older adults [22–25]. Numerous mechanisms have been suggested linking hypertension and physical function in late life, including impacts on the central nervous system, sarcopenia, inflammation, oxidative stress, among others [26]. These mechanisms are beyond the scope of this study but warrant further investigation in midlife.

The risk of heart disease and stroke rises drastically in late life [27] and accordingly, many studies have focused on physical function in relation to cardiovascular health in late life [28, 29]. However, the overall risk of cardiovascular events is rising among midlife women (a trend not seen among midlife men) [30] and midlife women have nearly twice the rate of stroke as midlife men [31]. If temporal trends of increasing prevalence of cardiovascular disease among women continue, especially at younger ages, it may also begin to play a larger role in disability development on the population level.

Midlife cardiovascular disease may play a critical role in identifying high-risk women for disability. Post-stroke rehabilitation is effective in improving physical function and reducing subsequent morbidity [32]. However, issues with mobility remain one of the most common long-term complications reported by patients in the years following a stroke [33]. Physical activity levels are generally low among even high-functioning stroke survivors, potentially contributing to further declines in physical function [34]. For heart disease, exercise-based cardiac rehabilitation is beneficial for improving fitness and health [35], and improvements in physical function have also been noted [36]. We do not have information in the current study as to whether participants who reported a stroke or heart disease were in cardiac rehabilitation, and therefore cannot determine if our results are reflective of patients who completed these programs. Still, uptake and completion of cardiac rehabilitation programs is low, and improvements in participation are needed [37].

We found that prevalent osteoporosis was related to worse initial physical function (nearly 12% lower than women without osteoporosis), and prevalent depressive symptoms were related to slightly accelerated decline in physical function, though neither were related to accelerated decline when developed later. For osteoporosis, changes in function are likely more evident later in the disease process, as osteoporotic fractures greatly contribute to a downward trajectory in physical function. We adjusted for fracture history in this study, though acute declines in physical function may have been evident if we had modeled the fracture as a fixed change-point rather than osteoporosis itself. Worse physical function was evident initially at follow-up visit 4 for women with osteoporosis, which was somewhat surprising given their relatively young age (early 50s) and presumably short disease duration at that point. These cases may instead represent severe early disease, as women are not routinely screened for osteoporosis until age 65 unless they are at increased risk of disease. In older adults bone loss and muscle strength co-occur, providing a potential mechanism linking osteoporosis with physical function [38, 39] For depressive symptoms, we utilized cut-points from a commonly used tool for assessing depressive symptoms, as we did not have information on clinically diagnosed depression. Thus, our estimates do not reflect the true effect of developing clinical depression on change in physical function. Further, the relationship between physical function decline and depressive symptoms could be bi-directional, or depressive symptoms could develop as a result of declining physical function.

Prevalent cancer at visit 4 was not associated with worse or worsening physical function, though cancer development was associated with an accelerated decline in physical function. We included several subtypes but were not able to include the time since diagnosis for women who had reported history of cancer at follow-up visit 4. Though combining cancer types was necessary from an analytic perspective, the varying symptoms, biology, and treatment may lead to different effects on physical function based upon cancer types. Cancer types were not distinguished consistently over time in SWAN, and the low prevalence of cancer overall would make it difficult to detect differences due to small numbers even if we were able to distinguish them consistently. In previous studies in breast cancer, self-reported physical function has been found to initially (within the first year) decline among older breast cancer survivors, though many women ultimately regain function [40]. Potentially, some women who reported cancer at analytic baseline may have been beyond the time of their initial drop in physical function and had instead regained function, leading to no apparent differences.

Strengths of this study include the long follow-up of a well-characterized cohort of women transitioning from mid to late life, as well as the use of novel statistical methods to investigate trajectories of physical function with the development of a new condition. In terms of retention at each visit, we had at least 84% retention at each follow-up visit (ranging from 84.3 to 88.8%) except for follow-up visit 15, which had 78.6% retention. Though we were able to study women as they transitioned from midlife to early late life, future investigation to determine as to how physical function changes further as these women continue to age is warranted. Development of a chronic condition in midlife versus late life may signal more severe disease, may lead to even greater decline in physical function in late life. Additionally, this study utilized a well-studied, common measure of self-reported physical function. One future direction is to investigate changes in performance-based physical function in relation to chronic condition development, as self-report and performance-based physical function measures are considered to capture related, but distinct, aspects of physical function. Potentially, women who developed chronic conditions or experienced serious health events were more cognizant of changes in physical function as they were also coping with other changes in their health status.

Some limitations of this study should also be considered. First, though we were able to include eight specific chronic conditions, confirmatory work is needed regarding the effects of these conditions as many only approached statistical significance. Though we maintained retention rates above 75% at each visit and over 95.2% of participants contributing at least 4 observations, the participants who did not complete follow-up visits may have had the steepest declines in physical function, though these declines would be unmeasured due to missing visits. This would bias our results towards the null and could account for some of our marginally significant results. Further, this is not an exhaustive list of chronic conditions that could influence physical function. Future studies examining other conditions that were not captured in SWAN are needed. We utilized self-reported clinician diagnosis (except for depressive symptoms) for defining presence of these conditions for consistency between conditions. Reliance on self-reported clinician diagnosis could potentially lead to an underestimation of conditions that are often asymptomatic early in the disease process—including hypertension, diabetes, or osteoporosis. Including clinical markers of disease may allow for detecting changes in physical function associated with disease processes even if a formal diagnosis has not been made, which is a future direction of this work.. However, this methodology is used by other large epidemiologic studies, including the Behavioral Risk Factor Surveillance Survey, and validation studies have found reasonable accuracy between self-report and medical records [41].

Conditions were considered separately in this study, when many women have—or ultimately develop—multiple chronic conditions. As we’ve previously shown, physical function decline accelerates with multiple chronic conditions [12]. Determining whether different combinations of conditions interact synergistically leading to additional effects on physical function decline is also a logical future direction of this work and could be useful in informing future interventions focused on chronic conditions and physical function in midlife. The effects associated with each of the conditions in this analysis were generally small, though specific combinations of chronic conditions may reveal synergistic effects on physical function decline. Conversely, appropriate treatment and management of these conditions could lead to either slowing of physical function decline, or even improvements. In this analysis we considered conditions cumulatively, so differences in condition severity could account for the variability we saw in physical function.

## Conclusion

In summary, chronic conditions were common among this cohort of midlife women, and many women developed new conditions during this timeframe. This study extended previous literature indicating that new chronic conditions in midlife are associated with physical function worsening to show how different chronic conditions are associated with this decline. Initial prevalent conditions related to the musculoskeletal system were associated with worse physical function at analytic baseline, with some evidence of accelerated decline in physical function with osteoarthritis. Stroke and heart disease are less common than osteoarthritis in this age group, but the effects of these conditions on physical function show the need for a greater focus on cardiovascular health during midlife. Interventions targeting physical function and mobility disability prevention typically focus on older adults. However, women who develop chronic conditions during midlife may be at particular risk for poor physical function as they age, warranting disability prevention efforts focused on this population.

## Data Availability

SWAN provides access to public use datasets that include data from SWAN screening, the baseline visit and follow-up visits (https://agingresearchbiobank.nia.nih.gov/). Some, but not all, of the data used for this manuscript are contained in the public use datasets. A link to the public use datasets is also located on the SWAN web site: http://www.swanstudy.org/swan-research/data-access/. Investigators who require assistance accessing the public use dataset may contact the SWAN Coordinating Center at the following email address: swanaccess@edc.pitt.edu.
